# Obstructive Sleep Apnea and Hypertension in Adolescents: Effect on Neurobehavioral and Cognitive Functioning

**DOI:** 10.1155/2016/3950914

**Published:** 2016-06-16

**Authors:** Irina Madaeva, Olga Berdina, Vladimir Polyakov, Sergey Kolesnikov

**Affiliations:** ^1^Somnological Center and Laboratory of Pathophysiology, Scientific Center for Family Health and Human Reproduction Problems, Timiryazev Street 16, Irkutsk 664003, Russia; ^2^Somnological Center and Laboratory of Pediatrics and Neurophysiology, Scientific Centre for Family Health and Human Reproduction Problems, Timiryazev Street 16, Irkutsk 664003, Russia; ^3^Laboratory of Children Psychoneurosomatic Pathology, Scientific Center for Family Health and Human Reproduction Problems, Timiryazev Street 16, Irkutsk 664003, Russia; ^4^Laboratory of Pathophysiology, Scientific Center for Family Health and Human Reproduction Problems, Timiryazev Street 16, Irkutsk 664003, Russia

## Abstract

*Background*. There are limited published data in regard to the relationship between obstructive sleep apnea (OSA) and hypertension and neurobehavioral and mental status in adolescence. The aim of our study was to evaluate neurobehavioral patterns and cognitive functions in adolescents with hypertension according to absence or presence of OSA.* Methods*. This was a retrospective cohort study completed at the Scientific Center for Family Health and Human Reproduction Problems. Participants included adolescents aged 14–17 years and referred for 24-hour ambulance blood pressure monitoring (ABPM) and polysomnographic (PSG) studies between 2007 and 2009, inclusive.* Results*. 18 hypertensive OSA (the 1st group) and 20 hypertensive non-OSA adolescents (the 2nd group) were included in the study. Significant changes of neurobehavioral functioning in OSA patients were shown. Cognitive abilities also were impaired. Verbal and visual memory indexes and attention index were 2.1 and 2.2 times lower, accordingly, in the 1st group than in the 2nd group (*P* < 0.05). Speech index was significantly 2.8 times lower in OSA patients than in non-OSA patients (*P* < 0.05). In hypertensive OSA adolescents more significant Spearman correlations between classic sleep parameters and cognitive measures were found compared to patients without OSA.* Conclusions*. These results suggest that OSA is closely associated with neurobehavioral and cognitive functioning in hypertensive adolescents.

## 1. Introduction

Sleep related breathing disorder (SRBD) represents a spectrum of conditions ranging from habitual snoring to frank obstructive and/or central sleep apnea that occurs in all age groups. With the relatively recent establishment of the medical specialty of Paediatric Sleep Medicine, obstructive sleep apnea (OSA), in particular, has gained much attention [1, 2]. OSA is associated with sleep fragmentation, intermittent hypoxemia, and hypercapnia [3]. These factors may result in neurobehavioral and cognitive deficits and possibly permanent damage, especially if the insults occur during adolescence, a time of significant neural reorganization and development. OSA is associated with behavioral dysfunctions including aggression, impulsiveness, and decreased attention [4]. Additionally, adolescents with OSA display lower IQ scores and lower scores on tests of memory and other executive functions [5, 6]. The potential neurobehavioral effects of OSA are well documented in adults and children [7, 8] yet scientific understanding is significantly less developed for the transitional stage of adolescence [9].

It is known that OSA and cardiovascular diseases are closely associated in adults. There is evidence that people with OSA have chronic dysregulation of cardiovascular homeostasis, as demonstrated by daytime abnormalities in sympathetic nervous system function and heart rate variability [10, 11]. A prospective study from the Wisconsin Sleep Cohort was the first to provide persuasive evidence implicating OSA as a possible causal factor in hypertension [12]. Blood pressure (BP) level correlates with emotional status, behavioral patterns, and current mental state. At present hypertension proved to be a risk factor for memory and other cognitive functions disorders already in adolescence [13, 14]. There are limited published data in regard to the relationship between SRBD and hypertension and behavioral and mental status in adolescence.

Given this fact, the aim of this study was to evaluate behavioral patterns and cognitive functions in hypertensive adolescents according to absence or presence of OSA.

## 2. Materials and Methods

### 2.1. Data Collection

Hypertension in adolescents was defined as systolic BP and/or diastolic BP that is on three separate measurements at or above the 95th percentile [15]. In this study hypertension was verified by 24-hour ambulance blood pressure monitoring (ABPM) using a portable device Oscar 2 system OXFORD Medilog Prima (England) by standard method. Charts of adolescents with hypertension meeting study inclusion criteria referred for polysomnographic (PSG) studies (GRASS-TELEFACTOR Twin PSG (Comet) c as the amplifier 40 with an integrated module for sleep SPM-1 (USA)) at the Somnological Center of the Scientific Center for Family Health and Human Reproduction Problems during a 2-year period from 2007 to 2009, inclusive, were reviewed. Date of PSG and interpretation of PSG were generated. Obstructive apnea was defined as a reduction in airflow of ≥90% associated with continued abdominal and chest wall motion lasting 2-3 breaths in duration. Hypopnea was defined as a peak signal excursion drop by ≥30% of preevent baseline lasting 2-3 breaths in duration associated with EEG arousal and/or ≥3% drop in oxygen saturation. Lastly, OSA was defined as an overall apnea hypopnea index of greater than 5 per hour of total sleep time [16].

Study inclusion criteria were ABPM verified hypertension and males of 14–17 years old at the time of their PSG and psychological evaluation.

Study exclusion criteria were body mass index (BMI)≥ 30 kg/m^2^, acute illness, and exacerbation of chronic illness at the time of their PSG and psychological evaluation.

### 2.2. Psychological Evaluation

Investigation of neurobehavioral patterns was carried out with the use of Lichko's Pathocharacterologic Diagnostic Questionnaire (PCDQ). The examination of cognitive abilities included attention processes with Schulte tables, audioverbal features and visual-spatial memory by memorizing ten words and icons, peculiarities of speech and thinking with “60 words” and “classification of objects” techniques, and making the story on the subject [17].

### 2.3. Data/Statistical Analysis

All statistical tests were performed using Statistica v6.0 (StatSoft, USA). For descriptive statistics, continuous variables were summarized as mean ± standard deviation. Shapiro-Wilk test *W* is used when checking for normal distribution. Student's *t*-test is used when assessing the reliability of differences of mean values. The Spearman correlation was used in order to evaluate the relationship between sleep characteristics and neurobehavioral/cognitive measures. The critical level of significance was taken as 0.05.

This study was approved by the Local Ethical Committee and a written informed consent was obtained from all participants or their parents (in case of being a child under 15 years old) at the assessment.

## 3. Results

38 patients aged 14–17 years were included in the study. All patients were divided into 2 groups according to the American Academy of Sleep Medicine (AASM) criteria [16]. The 1st group consists of 18 hypertensive adolescents with OSA and the 2nd of 20 hypertensive patients without OSA.

Study group characteristics are shown in Table 1.

They were all (100%) boys. As expected, day systolic BP differed significantly for adolescents without OSA compared to hypertensive OSA patients, but night systolic BP and diastolic BP were significantly higher in adolescents with OSA compared to hypertensive non-OSA patients. OSA adolescents with hypertension had a significantly higher BMI than the hypertensive patients without OSA.

Table 2 shows the comparisons of macrostructural sleep parameters and scoring indexes between hypertensive adolescents with and without OSA. Many differences were found: sleep onset latency (SOL) and wakefulness after sleep onset (WASO) were shorter in the 1st group than in the 2nd group (*P* < 0.05). The percentages of sleep stage 2 (S2) of nonrapid eye movement (NREM) sleep were higher (*P* < 0.05) and slow wave sleep (SWS), rapid eye movement (REM) sleep, and sleep efficiency (SE) were lower (*P* < 0.05) in adolescents with OSA compared to hypertensive non-OSA patients. Arousal index and desaturation were significantly increased in hypertensive OSA patients.

Table 3 shows the neurobehavioral characteristics of examined patients according to absence or presence of OSA.

Differences between groups on measures of behavior and depression are shown. The hypertensive OSA group had significantly increased inclination to manipulative behavior and reaction of emancipation compared to hypertensive non-OSA group. It is necessary to emphasize that hypertensive adolescents with OSA have significantly increased level of aggression compared to hypertensive adolescents without OSA. However, hypertensive OSA adolescents reported significantly decreased symptoms of depression compared to hypertensive non-OSA adolescents.

At a following stage of psychological study we investigated the cognitive functioning of hypertensive adolescents and conducted comparative analysis of relevant parameters, taking into account absence or presence of OSA (Table 4).

Adolescents with hypertension and OSA have much worse remembered stimuli of different modality, made replacements of the words taken from the shown set, and made new words and collateral associations at reconstruction audioverbal and visual-spatial traces. The indexes of verbal and visual memory in the hypertensive OSA group were 2.1 and 2.0 times lower than in hypertensive non-OSA group (*P* < 0.05).

The hypertensive OSA adolescents have significantly poorer attention compared to hypertensive non-OSA patients.

The hypertensive OSA adolescents have significantly decreased ability of classification of subjects on signs (the analysis processes) and generalization of received information (the synthesis processes) compared to hypertensive adolescents without OSA. The specific type of thinking dominated, and efficiency at performance of appropriating tasks was initially low in the hypertensive OSA group.

The study of the speech activity had shown that in these adolescents the overall vocabulary and the ability of the association were broken. Generally, indexes of progress of thinking and speech were significantly 1.4 and 1.9 times lower than in hypertensive adolescents without OSA, accordingly.

**Figure 1:**
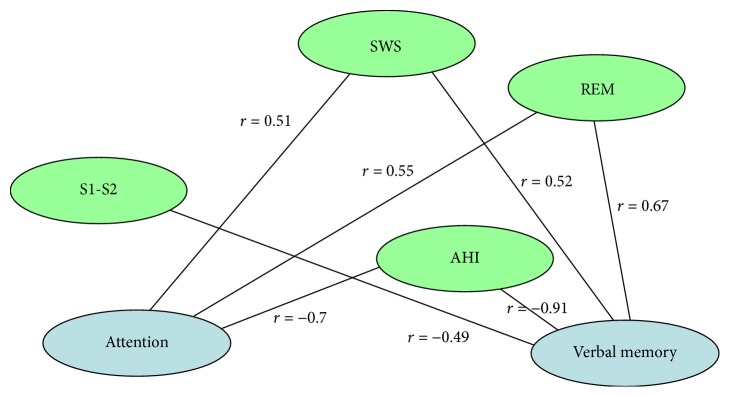
Spearman correlations between sleep parameters and cognitive measures are shown for hypertensive OSA adolescents. As can be seen, very high and statistically significant correlations were found between the verbal memory-AHI (*r* = −0.91, *P* < 0.05), the verbal memory-REM (*r* = 0.67, *P* < 0.05), and the attention-AHI (*r* = −0.7, *P* < 0.05).

**Figure 2:**
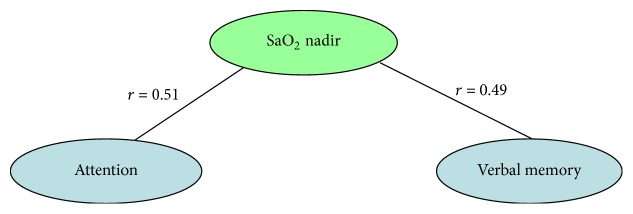
Spearman correlations between sleep parameters and cognitive measures are shown for hypertensive non-OSA patients. As can be seen, middle statistically significant correlations were found between the verbal memory-SaO_2_ nadir (*r* = 0.49, *P* < 0.05) and the attention-SaO_2_ nadir (*r* = 0.51, *P* < 0.05).

Spearman correlations were computed between sleep parameters (total sleep time (TST), SOL, S1, S2, SWS, REM, apnea hypopnea index (AHI), arousal index, and SpO_2_ nadir) and the results of the psychological tests (PSDQ and cognitive tests). Correlations between sleep parameters and the results of the psychological tests are illustrated in Figures 1 and 2. At the figures we showed only significant correlations for each group. No significant correlations between neurobehavioral measures were found in both groups. So, in the 1st group, verbal memory was positively correlated with SWS (*r* = 0.52, *P* < 0.05) and REM (*r* = 0.67, *P* < 0.05), but it was negatively correlated with S1-S2 (*r* = −0.49, *P* < 0.05) and AHI (*r* = −0.91, *P* < 0.05). Attention was positively correlated with SWS (*r* = 0.51, *P* < 0.05) and REM (*r* = 0.55, *P* < 0.05), but it was negatively correlated with AHI (*r* = −0.7, *P* < 0.05). In the 2nd group, of the different sleep parameters, only SpO_2_ nadir was positively significantly correlated with verbal memory (*r* = 0.49, *P* < 0.05) and attention (*r* = 0.51, *P* < 0.05).

## 4. Discussion

Adolescence is a time of rapid development of problem solving, information processing, judgment, and emotion regulation, as well as a time when significant behavioral health concerns such as depression or anxiety may begin [18]. Hence, factors affecting neurobehavioral functioning during this developmental stage may have a significant effect on neurologic outcomes. The neurodevelopment of hypertensive adolescents with OSA may be particularly vulnerable, which is highlighted by the findings of this study.

Based on prior research on OSA and adolescents [7, 9] as well as the physical development and reorganization of the adolescent brain, particularly the areas responsible for higher-level cognitive function (i.e., attention and executive function) and behavior, mood, and emotional regulation, we chose to focus our study on these aspects of daytime functioning.

This study found that, compared to hypertensive adolescents without OSA, adolescents with hypertension and OSA have neurobehavioral problems and impaired cognitive abilities.

So, the hypertensive OSA adolescents showed expressed inclination to not restrained aggressive and manipulative behavior and an increase of the reaction of emancipation compared to hypertensive non-OSA patients, who had greater stability and predictability in behavioral reactions. We suggest the given circumstance is the reflection of the formation of the center of pathologically strengthened excitation due to superfluous quantity of arousals in the cerebral cortex and, mostly, in its frontal lobes, during sleep with obstructive respiratory disorders.

It must be emphasized that adolescents with a combination of hypertension and OSA had also significant changes of cognitive functioning. In this group we did find more significant correlations between classic sleep parameters and cognitive measures (verbal memory and attention) compared to patients without OSA. We assume that the change of memory processes and a lot of significant correlations in hypertensive OSA patients indicates both impaired restoration of cognition within SWS and REM sleep and changes in functional state of the temporal-occipital and medial frontal lobes of the brain as a result of the hypoxia that occurs every night during episodes of apnea/hypopnea.

## 5. Conclusion

In summary, hypertensive adolescents with OSA showed impaired neurobehavioral and cognitive functioning, compared to hypertensive non-OSA patients. The results of this study suggest that pediatricians should consider OSA as a contributing etiologic factor for behavior and school performance problems in adolescents with hypertension. Given the deficits identified in this study, we speculate that untreated OSA during adolescence may lead to neurobehavioral and cognitive deficits in adulthood. Future research should evaluate changes following successful treatment of OSA in hypertensive adolescents and longitudinal outcomes in adulthood.

## Figures and Tables

**Table 1:** Participant demographic and blood pressure characteristics in hypertensive adolescents with and without OSA.

	Hypertension with OSA	Hypertension without OSA	*P*
*N*	18	20	
Age (years)	16.5 ± 0.3	16.1 ± 0.2	NS
Males, *N* (%)	18 (100)	20 (100)	
BMI (kg/m^2^)	25.9 ± 2.7	23.1 ± 1.5	**<0.05**
24 h BP monitoring			
Day systolic BP (mm Hg)	131 ± 3.5	142 ± 3.2	**<0.05**
Day diastolic BP (mm Hg)	72 ± 2.6	80 ± 2.1	NS
Night systolic BP (mm Hg)	138 ± 2.1	115 ± 1.8	**<0.05**
Night diastolic BP (mm Hg)	75 ± 1.5	63 ± 1.3	**<0.05**

Data shown as *N* (%) or mean ± standard error. Significant differences are indicated by bold print.

BMI: body mass index; BP: blood pressure.

**Table 2:** Comparison of sleep scoring parameters found in the 2 groups of study subjects.

	Hypertension with OSA	Hypertension without OSA	*P*
TST, min	471.3 ± 50.9	468.1 ± 38.8	NS
SOL, min	7.8 ± 2.8	27.2 ± 9.3	**<0.05**
WASO, min	12.7 ± 4.2	19.1 ± 2.1	**<0.05**
SE, %	72.1 ± 3.2	89.7 ± 2.5	**<0.05**
S1, %	2.5 ± 1.5	2.3 ± 1.7	NS
S2, %	71.2 ± 9.1	49.2 ± 6.7	**<0.05**
SWS, %	12.4 ± 2.1	16.9 ± 1.5	**<0.05**
REM, %	13.4 ± 3.1	30.7 ± 3.5	**<0.05**
AHI, /h TST	13.7 ± 1.5	1.1 ± 0.5	**<0.05**
Arousal index, /h TST	28.6 ± 0.4	18.2 ± 0.3	**<0.05**
SpO_2_ nadir, %	90.8 ± 1.1	97.1 ± 0.5	**<0.05**

Data shown as *N* (%) or mean ± standard error. Significant differences are indicated by bold print.

TST: total sleep time; SOL: sleep onset latency; WASO: wakefulness after sleep onset; SE: sleep efficiency; S1, S2: sleep stages 1 and 2; SWS: slow wave sleep; REM: rapid eye movement sleep; AHI: apnea hypopnea index; SpO_2_ nadir: nadir oxyhemoglobin saturation by pulse oximetry.

**Table 3:** Neurobehavioral characteristics of examined patients according to absence or presence of OSA.

	Hypertension with OSA (*n* = 18)	Hypertension without OSA (*n* = 20)	*P*
Inclination to manipulative behavior, points	44.4 ± 1.28	37.9 ± 1.55	**<0.05**
Reaction of emancipation, points	33.5 ± 1.58	20.0 ± 1.74	**<0.05**
Level of aggression, points	29.7 ± 1.31	14.7 ± 1.52	**<0.05**
Level of depression, points	15.5 ± 1.7	40.2 ± 1.7	**<0.05**

Data shown as mean ± standard error. Significant differences are indicated by bold print.

**Table 4:** Cognitive functioning of hypertensive adolescents according to absence or presence of OSA.

	Hypertension with OSA (*n* = 18)	Hypertension without OSA (*n* = 20)	*P*
Verbal memory, points	6.4 ± 1.18	11.9 ± 1.27	**<0.05**
Visual memory, points	15.9 ± 1.25	27.0 ± 1.34	**<0.05**
Attention, points	24.7 ± 1.44	45.7 ± 1.13	**<0.05**
Verbal thinking, points	17.5 ± 1.3	25.2 ± 1.7	**<0.05**
Speech, points	11.5 ± 1.25	19.5 ± 1.2	**<0.05**

Data shown as mean ± standard error. Significant differences are indicated by bold print.
